# Ischaemic Cardiomyopathy Secondary to Asymptomatic Coronary Artery Disease: A Case Report

**DOI:** 10.7759/cureus.68766

**Published:** 2024-09-06

**Authors:** Gedoni Eni, Allison Ramirez, Roshan Faiz, Jhiamluka Solano

**Affiliations:** 1 Internal Medicine, Scunthorpe General Hospital, Scunthorpe, GBR; 2 Internal Medicine, Hospital General San Francisco, Olancho, HND; 3 Education Committee, Academy of Medical Educators, Cardiff, GBR; 4 Cardiology, Scunthorpe General Hospital, Scunthorpe, GBR

**Keywords:** heart failure with reduced ejection fraction, ischaemic heart disease, ischemic dilated cardiomyopathy, mri cardiac, silent myocardial ischaemia

## Abstract

Ischaemic cardiomyopathy (ICM) represents a common complication of coronary artery disease (CAD). Ischaemia causes ventricular remodelling, leading to an irreversible loss of myocardial tissue and adequate contractility, primarily affecting the left ventricular ejection fraction (LVEF). We present the case of a 46-year-old male known as hypertensive presented to the hospital with a five-week history of progressive exertional dyspnoea, bilateral lower limb oedema subsequently involving his scrotum and penis. He reported reduced oral intake and occasional palpitations but denied chest pain, cough, fever, or haemoptysis. He had no personal history of cardiac disease, recent travels, or recreational drug use. Notably, he consumed approximately 12 units of alcohol weekly and was a non-smoker. On admission, he was treated for new-onset heart failure, and initial investigations showed acute kidney injury, raised troponin, and brain natriuretic peptide (BNP), and chest X-ray showed an enlarged heart size (cardiothoracic ratio (CTR), 0.56) with moderate right pleural effusion. Echocardiography revealed a severely dilated left ventricle with severely impaired systolic function (LVEF 16%), bi-atrial dilatation, borderline dilated right ventricle with impaired systolic function, and moderate tricuspid regurgitation. Cardiac MRI showed that the left ventricle was severely dilated with severely impaired systolic function with nonviable mid to apical inferior and inferoseptal transmural post-ischaemic scar with associated hypokinesia. Ischaemic cardiomyopathy may vary from asymptomatic to severely symptomatic, commonly when symptomatic patients will present with anginal chest pain and dyspnoea on exertion. In contrast, asymptomatic patients can sometimes have up to 80% of transient ischaemic events with no chest pain or associated symptoms. This case underscores the importance of considering asymptomatic coronary artery disease in clinical practice and highlights the need for novel interventions and markers for early ischemia detection.

## Introduction

Ischaemic cardiomyopathy (ICM) is a well-recognised consequence of coronary artery disease (CAD), characterised by ischaemia-induced ventricular remodelling, which leads to irreversible myocardial tissue loss and impaired contractility, predominantly affecting left ventricular ejection fraction [1]. This pathological process results in ventricular dilation and the subsequent development of heart failure symptoms. Despite the link between ischaemia and dilated cardiomyopathy (DCM), the European Society of Cardiology (ESC) excluded ICM from its 2007 classification of cardiomyopathies because of the need to create a more comprehensive clinically oriented classification based on myocardial disorders grouped according to ventricular morphology and function [2].

Clinically, the presentation of ICM can vary from asymptomatic to severely symptomatic manifestations. Symptomatic patients typically present with anginal chest pain and exertional dyspnoea, while asymptomatic individuals may experience transient ischaemic events without classical symptoms of CAD [3]. The diagnostic process for ICM can be challenging, particularly in asymptomatic patients, due to overlapping clinical conditions and non-specific presentations. Nonetheless, standard cardiovascular investigations including electrocardiogram, echocardiography, coronary angiography, and cardiac magnetic resonance imaging (MRI) are integral to the diagnostic approach [4]. Cardiac MRI, utilising late gadolinium enhancement, can help in differentiating ischaemic from non-ischaemic cardiomyopathies [1,3,4].

Management of ICM focuses on adhering to standardised heart failure treatment protocols with emphasis on addressing modifiable risk factors like smoking and providing appropriate medical therapy for comorbidities such as hypertension, diabetes mellitus, obesity, and hyperlipidaemia [5]. The complexity of ischaemic cardiomyopathy, particularly in asymptomatic patients, necessitates a comprehensive approach to both diagnosis and treatment to optimise patient outcomes and limit further disease progression.

## Case presentation

A 46-year-old male presented with a five-week history of progressive exertional dyspnoea and bilateral lower limb oedema, extending to involve the scrotum and penis. In the week preceding hospitalisation, he developed paroxysmal nocturnal dyspnoea. He also reported reduced oral intake and occasional palpitations but denied chest pain, cough, fever, or haemoptysis. His medical history was significant for hypertension, managed with ramipril and amlodipine, and a weekly alcohol consumption of approximately 12 units. He had no history of angina, rheumatic fever, recent travel, surgery, or recreational drug use, and he was a non-smoker.

**Figure 1:**
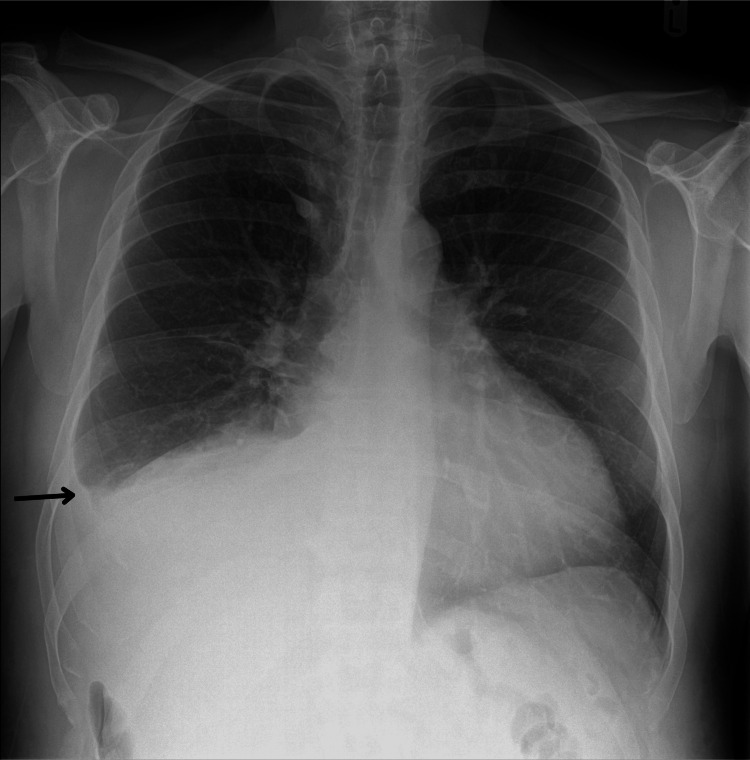
PA radiograph of the thorax on admission. The PA radiograph of the thorax shows blunting (arrow) of the right costophrenic angle. PA: posteroanterior.

On examination, heart rate was 103 beats per minute, respiratory rate was 18 cycles per minute, and oxygen saturation was 96% on room air. Elevated jugular venous pressure (5cm) and bi-basal crackles were noted on lung auscultation. The abdominal examination revealed abdominal distension, hepatomegaly, and ascites. Laboratory investigations showed hyponatremia (129 mmol/L), acute kidney injury with marked azotaemia and uraemia (19.8 mmol/L) and creatinine (198 µmol/L), reflecting a marked deviation from baseline normal renal function. Additionally, alanine aminotransferase was markedly elevated (2022 IU/L) from a normal baseline with increased bilirubin (38 µmol/L), troponin (159 ng/L), and brain natriuretic peptide (BNP) levels exceeding 15,000 ng/L. Chest X-ray demonstrated cardiomegaly (cardiothoracic ratio of 0.56) and moderate right pleural effusion (see Figure 1). Initial ECG showed T-wave inversion in leads V4 to V6, aVL, and I.

**Figure 2:**
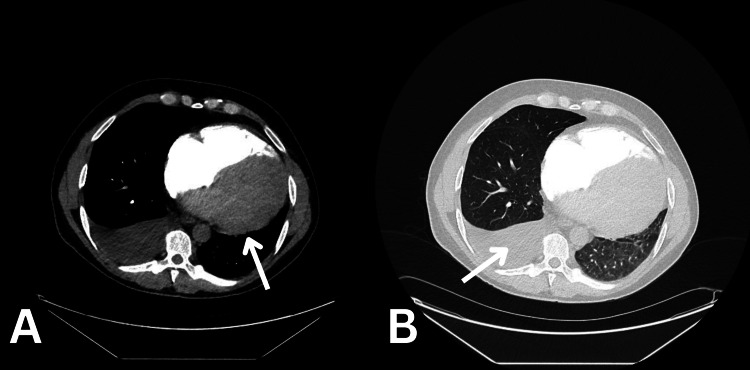
CT-PA soft tissue (A) and pulmonary (B) view. A: The arrow highlights the enlarged and globular heart; B: The arrow points to the right pleural effusion. Note: Contrast reflux is noted to enter into the inferior vena cava and hepatic veins. PA: pulmonary angiogram.

**Figure 3:**
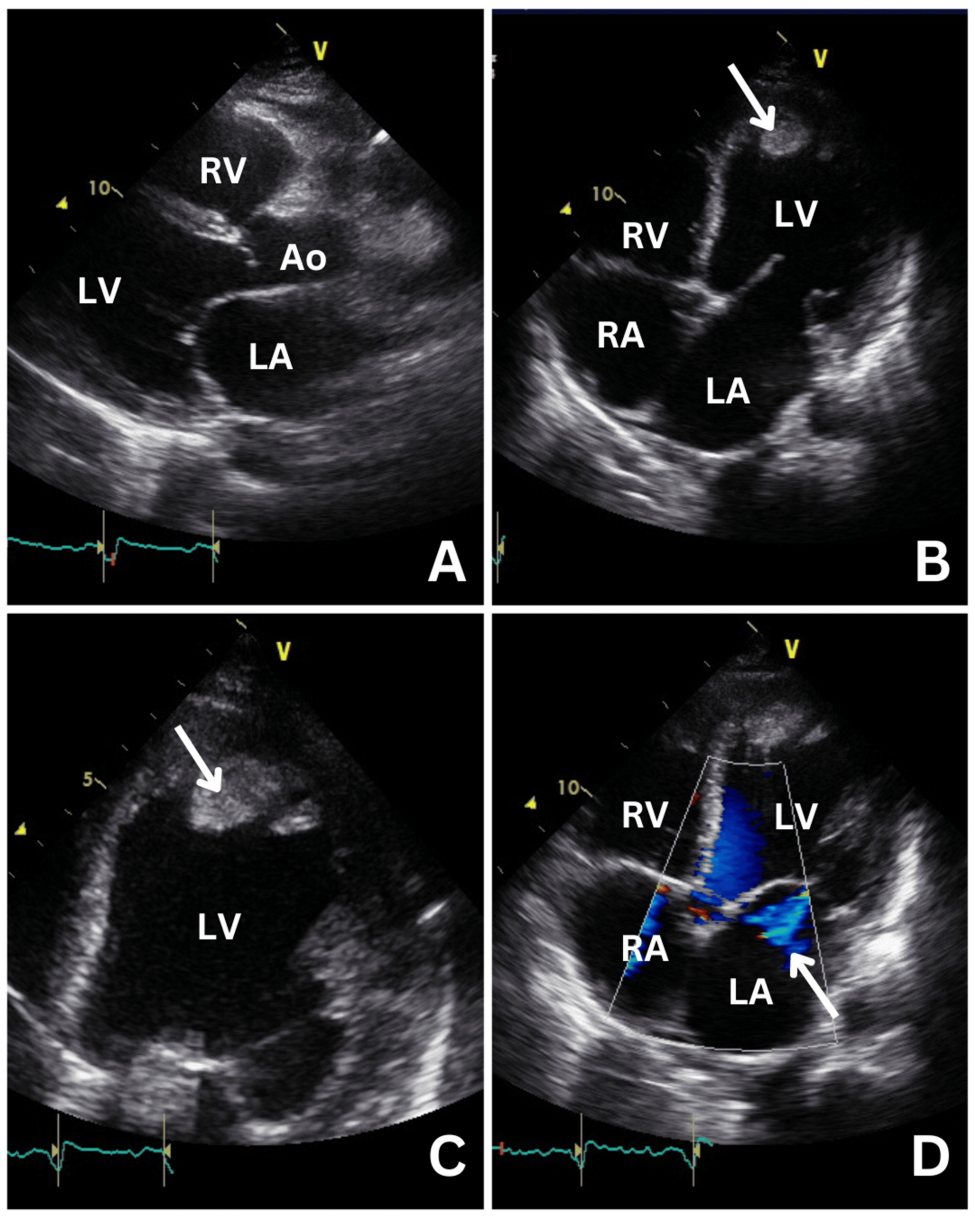
Transthoracic echo with labelled chambers. A. Parasternal long axis showing a dilated LV (left ventricle); B. apical four window showing LV apical thrombus (arrow) and dilated LV and LA (left atrium); C. focused view of LV apical thrombus (arrow); and D. functional mitral valve regurgitation colour Doppler eccentric jet (arrow) secondary to dilated LV. RV: right ventricle, RA: right atrium, Ao: aortic valve.

Initial assessment pointed towards acute heart failure with hepatic congestion and acute kidney injury. Differential diagnoses included heart failure secondary to myocarditis, cardiomyopathy or pericarditis, and pulmonary embolism. Computed tomography pulmonary angiography (CTPA) ruled out pulmonary embolism but showed a significantly enlarged heart with contrast reflux into the inferior vena cava and hepatic veins, alongside a right-sided pleural effusion (Figure 2). Echocardiography revealed a severely dilated left ventricle (LV) with severely impaired systolic function (ejection fraction of 16%), a moderate-sized apical thrombus, bi-atrial dilatation, borderline dilated right ventricle (RV) with impaired systolic function, and moderate tricuspid regurgitation (TR) (Figure 3). These findings prompt the initiation of therapeutic dose of low molecular weight heparin.

**Figure 4:**
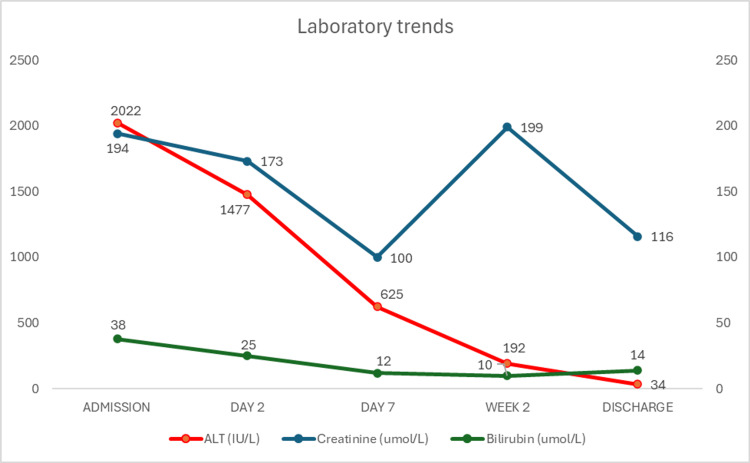
Blood investigations conducted during admission, post-initial treatment, and before discharge. ALT: alanine aminotransferase.

Following adequate diuresis, the patient’s liver and renal function normalised during hospitalisation (Figure 4). Initial management was challenging, but a combination of heart failure medications, including bumetanide, bisoprolol, dapagliflozin, ivabradine, sacubitril/valsartan, and spironolactone, led to a satisfactory clinical response.

**Figure 5:**
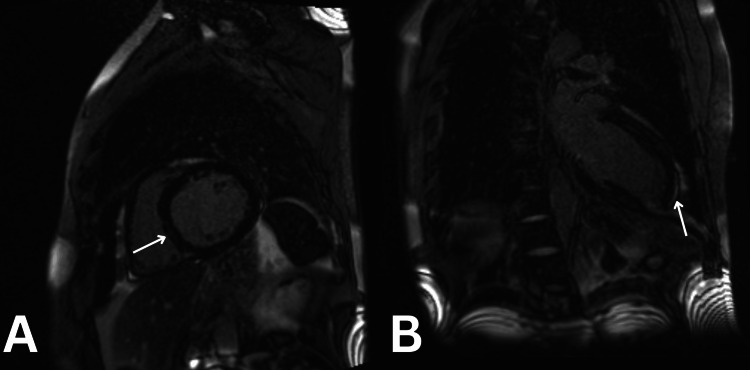
Cardiac MRI with BLG (basic late gadolinium) and thrombus sequences (perfusion and early gadolinium) technique. A. Inferoseptal transmural post-ischaemic scar (arrow); and B. apical inferior transmural post-ischaemic scar (arrow).

Before discharge, cardiac MRI with basic late gadolinium (BLG) enhancement and thrombus sequences revealed a severely dilated left ventricle with severely impaired systolic function, a mildly dilated right ventricle with severely impaired systolic function, nonviable mid to apical inferior and inferior-septal transmural post-ischaemic scarring with associated hypokinesia, mild bi-atrial dilation, moderate central functional mitral valve regurgitation, and no evidence of cardiac thrombus (Figure 5). At a three-month follow-up, repeat echocardiography showed an improved ejection fraction of 41% following optimal heart failure therapy. The patient was scheduled for an outpatient diagnostic angiogram.

## Discussion

Ischemic cardiomyopathy (ICM) is characterised by diminished cardiac function due to a chronic mismatch between myocardial oxygen supply and demand, ultimately leading to ventricular failure. Clinical manifestations of ICM vary widely, ranging from stable chronic angina to sudden cardiac death, primarily affecting middle-aged and elderly populations [3,4]. Clinical and radiographically findings presented by the patient were consistent with heart failure, e.g., raised jugular venous pressure (JVP), bibasal crackles, palpitations, pleural effusion, and contrast reflux into the inferior vena cava and hepatic veins support the diagnosis of heart failure or provide insights into the heart's functional status. A thorough patient history often reveals evidence of prior myocardial infarctions and chronic coronary artery disease [4]; however, in this case, the patient reported no such history. Additionally, considering the patient’s social and family history could suggest an underlying aetiology. The patient presented with heart failure symptoms, accompanied by a rapid decline in ventricular function, and despite hypertension being the only identified risk factor for CAD, an ischaemic aetiology remained a consideration among the differential diagnosis because of the suspected apical thrombus found on echocardiography.

Laboratory tests revealed significantly elevated alanine aminotransferase (ALT) levels, likely due to congestive hepatopathy from increased venous pressure transmitted to the hepatic veins and sinusoids. This was consistent with the clinical findings of hepatomegaly and ascites. The observed hepatic dysfunction aligns with the findings reported by Nikolaou and colleagues [6] but differs from other studies suggesting a predominance of cholestatic liver injury in heart failure [7,8]. Notably, the patient was asymptomatic from a hepatic perspective, and abdominal ultrasound did not reveal structural liver abnormalities. The liver enzyme derangement may also reflect ischemic hepatitis, where reduced hepatic blood flow leads to centrilobular necrosis and elevated bilirubin levels [6,7]. The improvement in liver enzymes and bilirubin following intravenous diuretic therapy, which reduced central venous pressure, supports congestive hepatopathy as the primary underlying mechanism of liver injury. Other potential causes, such as hypoxic hepatitis ('shock liver') and medication-induced hepatic dysfunction, were deemed less likely. This clinical response highlights the interplay between cardiac function and hepatic health, emphasising the importance of correcting systemic congestion to preserve liver function and prevent progression to cardiac cirrhosis.

The development of acute kidney injury in the context of severe left ventricular dysfunction and clinical signs of congestion was anticipated. Existing literature [8-10] describes several pathophysiological mechanisms underlying this association, including inflammatory, hemodynamic, and neurohormonal processes, such as activation of the renin-angiotensin-aldosterone system (RAAS) and diuretic-induced fluid shifts or hypovolemia. This complex interplay extends beyond the heart and kidneys alone; the hepatorenal reflex acts as an additional trigger of renal dysfunction in congestive hepatopathy [8]. After initiating intravenous furosemide, regular blood tests showed increased serum creatinine and a corresponding decrease in the estimated glomerular filtration rate (eGFR). However, treatment continued, and renal function eventually improved. Diuretic therapy can exacerbate creatinine rise through mechanisms including hypovolemia, decreased renal perfusion, RAAS activation, and direct nephrotoxicity [11,12]. Therefore, the transient rise in serum creatinine was not concerning, but these patients require active monitoring of renal function with diuretics modified or discontinued if renal impairment significantly worsens.

This diagnosis of ischaemic cardiomyopathy was established based on clinical presentation, echocardiogram, and MRI findings, suggesting type 1 silent myocardial ischaemia (SMI) leading to dilated cardiomyopathy. Silent myocardial ischaemia presents a clinical challenge with no clear treatment guidelines [13,14]. The mechanism of silent myocardial ischemia remains poorly understood [13], but theories include altered pain perception, higher pain thresholds, elevated endorphin levels, cytokines interfering with pain pathways, and brief ischemic episodes going unnoticed [1,3,13,14]. The prevalence of SMI is difficult to ascertain but may be rising in the younger population and reportedly ranging between 10% and 30% in higher-risk individuals such as elderly patients or those with diabetes mellitus or end-stage renal disease [3]. The index patient fits the type I SMI profile based on the absence of symptoms and prior CAD history (Table 1).

**Table 1:** Silent MI classification. MI: myocardial infarction. Extracted from Theofilis et al. [14].

Type	Definition
I	Occurring in patient with asymptomatic coronary artery disease without collateral anginal symptoms.
II	Manifesting in patients with a history of myocardial infarction.
III	Observed in patients with concurrent or collateral manifestations of chronic stable angina, unstable angina, and vasospastic angina.

The patient was initially too unwell to undergo coronary angiography to assess the coronary arteries objectively. Current guidelines [15] recommend non-invasive stress testing in patients with a high-risk medical history, including echocardiography and cardiac MRI (with T1, T2 sequences, and late gadolinium enhancement), as performed in this case. Based on individual case assessment and risk-benefit analysis, further investigations such as computed tomography, coronary angiography (CTCA) and positron emission tomography (PET) could be considered. The role of myocardial scar tissue in cardiac function is worth noting, as it can lead to a stiffened myocardial matrix or impaired electric conductance [1].

Over the years, various classification systems for cardiomyopathies have been proposed, shaped by the evolving spectrum of the disease, advancements in medical science, and novel insights into its complex aetiopathogenesis. The 2007 European Society of Cardiology (ESC) introduced a classification system based on morphological and functional phenotypes, further subdividing these into familial and non-genetic subclasses [2]. This system was proposed as an update to the prior classification by the World Health Organization (WHO) and the International Society and Federation of Cardiology (ISFC) in 1995 [16]. Notably, the ESC authors [2] excluded ventricular dysfunction due to coronary artery disease and valvular disease from their classification of cardiomyopathies, arguing that these conditions, included in the 1995 classification, involve distinct pathophysiological mechanisms separate from other cardiomyopathies. The pathogenesis of ischaemic cardiomyopathy is fundamentally linked to coronary artery disease, which triggers a cascade of adverse metabolic, inflammatory, neurohormonal, and structural changes, culminating in cell death, fibrosis, left ventricular enlargement, and dilatation as part of the cardiac remodelling process [1,2,15,16].

Management of ischaemic cardiomyopathy is grounded in the principles of patient education, lifestyle modification, goal-directed medical therapy, and revascularisation, but treatment must be individualised, balancing the benefits and potential risks [15]. The benefits of revascularization with coronary artery bypass grafting (CABG) or percutaneous coronary intervention (PCI) regarding all-cause mortality and improved patient outcomes remain contentious. Two landmark trials, the STICH (Surgical Treatment for Ischemic Heart Failure) trial and REVIVED (Randomized Evaluation of PCI Versus Optimal Medical Therapy for Ischemic Left Ventricular Dysfunction) trial, compared long-term patient outcomes following revascularisation [17,18]. The STICH trial demonstrated a reduction in common modes of death, particularly sudden death, fatal myocardial infarction (MI), and acute pump failure in the CABG arm. However, there were observed increases in post-procedural mortality, highlighting the significant risks associated with this intervention [17]. On the other hand, heart failure with reduced ejection fraction management is known to have a positive prognostic factor, improvement in heart function and symptoms, as evidenced by the chosen heart failure medications prescribed for our patient and follow-up echocardiography [12].

Conversely, the REVIVED trial found no significant reduction in all-cause mortality or heart failure hospitalisations with PCI compared to optimal medical therapy. However, it did suggest modest improvements in quality of life and LV ejection fraction [18]. In the index patient, a cardiac MRI revealed areas of non-viable myocardium, which would not benefit from revascularization. However, an angiogram is still recommended to define the extent of coronary artery disease more precisely. The decision for revascularisation should consider the extent of coronary disease, comorbidities, and overall clinical context, ideally discussed in a multidisciplinary team meeting [15]. Optimal medical therapy remains the cornerstone of treatment for patients with ischaemic cardiomyopathy. It should include medications known to confer mortality benefits, such as beta-adrenergic blockers, angiotensin-converting enzyme inhibitors/angiotensin receptor blockers, statins, antiplatelet agents, mineralocorticoid receptor antagonists, and angiotensin receptor-neprilysin inhibitors [1,4,15-18].

Despite the advancements in diagnostics and risk stratification systems, identifying patients with asymptomatic CAD and no risk factors remains a challenge. Most patients with asymptomatic disease and risk factors will be initially detected during imaging tests to refine cardiovascular risk assessment. The ESC in 2024 recommends against systematic screening as it has not proven to affect CVD outcomes. However, opportunistic screening is recommended using the Systematic Coronary Risk Estimation 2 (SCORE 2) and its variations during follow-up for other reasons, e.g., hypertension, hyperlipidaemia, or diabetes mellitus [19]. Nonetheless, it is worth highlighting the need to develop and study further imaging techniques and protocols with new biomarkers to detect early ischaemia.

## Conclusions

We described a case of silent myocardial ischaemia progressing to ischaemic cardiomyopathy in a young patient without typical risk factors for asymptomatic coronary artery disease. This case highlights the complex pathophysiology and clinical challenges in managing ischaemic cardiomyopathy. The patient’s initial asymptomatic course, followed by progressive heart failure symptoms, emphasises the critical need for early detection and intervention in coronary artery disease to prevent such progression.

This case underscores the importance of considering asymptomatic coronary artery disease in clinical practice and highlights the need for novel interventions and markers for early ischemia detection. Such measures are vital for improving detection, halting myocardial damage, and ultimately enhancing patient outcomes. Future research should focus on understanding the long-term outcomes of various therapeutic strategies in ischaemic cardiomyopathy to inform clinical decision-making better.
